# Retraction Note: Effect of hCMSCs and liraglutide combination in ALI through cAMP/PKAc/β-catenin signaling pathway

**DOI:** 10.1186/s13287-023-03474-6

**Published:** 2023-09-29

**Authors:** Yun Feng, Linlin Wang, Xiaoying Ma, Xiaotong Yang, Ocholi Don, Xiaoyan Chen, Jieming Qu, Yuanlin Song

**Affiliations:** 1 Department of Pulmonary Medicine, Zhongshan Hospital, Fudan University, Shanghai, 20003 China; 2 Department of Respiration, Ruijin Hospital, School of Medicine, Shanghai Jiao Tong University, Shanghai, 20025 China; 3 Institute of Respiratory Diseases, School of Medicine, Shanghai Jiao Tong University, Shanghai, 20025 China; 4 Shanghai Respiratory Research Institute, Shanghai, 20003 China; 5 State Key Laboratory of Bioreactor Engineering & Shanghai Key Laboratory of New Drug Design, School of Pharmacy, East China University of Science and Technology, Shanghai, 200237 People’s Republic of China; 6 Department of Pathology, Ruijin Hospital, School of Medicine, Shanghai Jiao Tong University, Shanghai, 20025 China; 7 Department of Pulmonary Medicine, Zhongshan Hospital, Qingpu Branch, Fudan University, Shanghai, 201700 China; 8 National Clinical Research Center for Aging & Medicine, Huashan Hospital, Fudan University, Shanghai, 200040 China

**Retraction Note: Stem Cell Research & Therapy (2020) 11:2** **https://doi.org/10.1186/s13287-019-1492-6**

The Editors-in-Chief have retracted this article after concerns were raised about potential image re-use between Fig. 4D and 5D and the potential partial overlap between Fig. 6A and Supplementary Fig. 4A. The authors were unable to provide raw data for Figs. 4 and 5. The raw images provided for Fig. 6A and Supplementary Fig. 4A contained further partial image overlaps between different experimental conditions. The authors were also unable to share evidence of ethics approval. Therefore, the Editors have lost confidence in the data presented here.

Yuanlin Song agrees to this retraction. Yun Feng and Xiaotong Yang do not agree to this retraction. Linlin Wang, Xiaoying Ma, Ocholi Don, Xiaoyan Chen and Jieming Qu have not responded to any correspondence from the editor/publisher about this retraction.

